# Interleaflet Coupling, Pinning, and Leaflet Asymmetry—Major Players in Plasma Membrane Nanodomain Formation

**DOI:** 10.3389/fcell.2016.00155

**Published:** 2017-01-10

**Authors:** Toyoshi Fujimoto, Ingela Parmryd

**Affiliations:** ^1^Department of Anatomy and Molecular Cell Biology, Nagoya University Graduate School of MedicineNagoya, Japan; ^2^Science for Life Laboratory, Medical Cell Biology, Uppsala UniversityUppsala, Sweden

**Keywords:** membrane nanodomains, molecular pinning, liquid ordered domains, interleaflet coupling, membrane asymmetry, lipid interdigitation, phospholipid distribution

## Abstract

The plasma membrane has a highly asymmetric distribution of lipids and contains dynamic nanodomains many of which are liquid entities surrounded by a second, slightly different, liquid environment. Contributing to the dynamics is a continuous repartitioning of components between the two types of liquids and transient links between lipids and proteins, both to extracellular matrix and cytoplasmic components, that temporarily pin membrane constituents. This make plasma membrane nanodomains exceptionally challenging to study and much of what is known about membrane domains has been deduced from studies on model membranes at equilibrium. However, living cells are by definition not at equilibrium and lipids are distributed asymmetrically with inositol phospholipids, phosphatidylethanolamines and phosphatidylserines confined mostly to the inner leaflet and glyco- and sphingolipids to the outer leaflet. Moreover, each phospholipid group encompasses a wealth of species with different acyl chain combinations whose lateral distribution is heterogeneous. It is becoming increasingly clear that asymmetry and pinning play important roles in plasma membrane nanodomain formation and coupling between the two lipid monolayers. How asymmetry, pinning, and interdigitation contribute to the plasma membrane organization is only beginning to be unraveled and here we discuss their roles and interdependence.

## Membrane asymmetry

**Figure 1:**
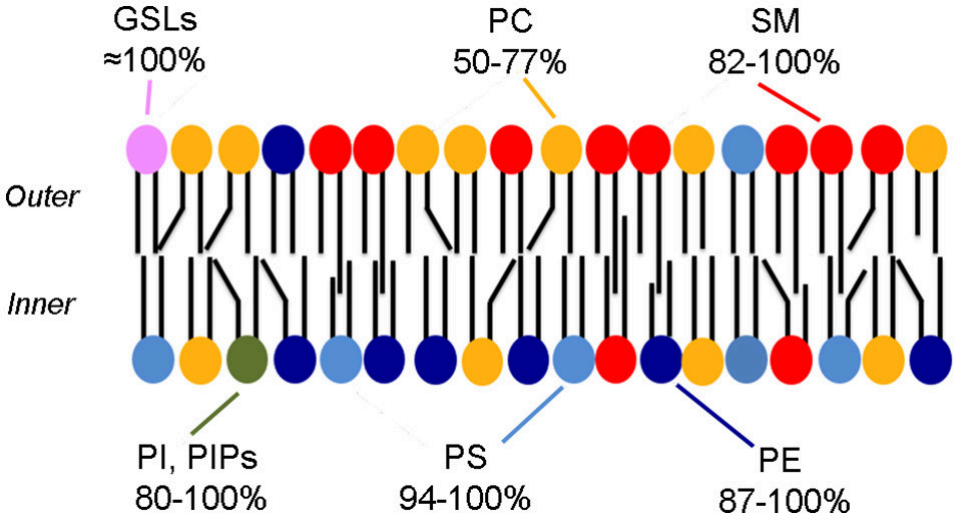
**Phospholipid asymmetry in the erythrocyte membrane**. The colors indicate the asymmetric distribution of phospholipids. The range in percentages indicates the amounts present in the preferred leaflet [all but the GSLs (Lingwood, 2011) from Table 1 from Zachowski (1993)]. Note that most phospholipids are also likely to be present in the less favored leaflet, albeit in a small amounts. Cholesterol is also a major component of the membrane but is not shown.

Already in the early 1970's it was known that the human erythrocyte membrane displays leaflet asymmetry in the phospholipid composition (Bretscher, 1972; Verkleij et al., 1973) with most phosphatidylcholine (PC) and sphingomyelin (SM) present in the outer leaflet, whereas phosphatidylserine (PS), phosphatidylethanolamine (PE), and phosphatidylinositol (PI) are in the inner leaflet (Figure 1). This architecture has often been regarded as a prototype of the plasma membrane of mammalian cells.

In recent years methods to produce asymmetric phospholipid membranes have been developed and together with sophisticated molecular simulation techniques, they have provided compelling evidence that lipids in one leaflet of the membrane can influence molecular diffusion and domain formation of lipids in the other leaflet without protein intervention, i.e., interleaflet coupling. In these studies, model membranes with perfect phospholipid distribution asymmetry are often used, for example with PC and/or SM in one of the leaflets and PS, PE, and/or PI confined to the opposite leaflet. However, in natural membranes, no phospholipid species is likely to distribute exclusively to only one leaflet and also small “imperfections” in asymmetry might have a significant impact in some membrane properties as we discuss below.

To better understand the properties of natural membranes, it is important to establish the degree of asymmetric phospholipid distribution. We will therefore summarize what is known about the distribution of individual lipids in the plasma membrane leaflets of mammalian cells. Here we have to emphasize two points. The first is the divergence between cell types: the most well-studied human erythrocyte membrane and the plasma membrane of other cells. The second is the degree of asymmetry: an asymmetric distribution of a particular lipid means that the distribution between the two leaflets is not 50:50. It could be 60:40, but it could also be nearly 100:0, and the distribution or any change thereof is likely to influence membrane properties. Using the lipid distribution data from natural membranes, we should be able to generate realistic models to explain the role of the asymmetric lipid distribution in cellular processes taking place in membranes.

## Lipid asymmetry in the plasma membrane

### Phosphatidylcholine

PC in the human erythrocyte membrane is predominantly found in the outer leaflet, and the proportion of PC in the outer leaflet was estimated to be 76–78% (Verkleij et al., 1973; van Meer et al., 1981). This level of asymmetry does not appear to exist in other cell types or even in erythrocytes of other species. For example, the proportion of PC in the outer leaflet of mouse, rat, and monkey erythrocyte membranes was reported to be 50% (Rawyler et al., 1985), 62–63% (Renooij et al., 1976; Crain and Zilversmit, 1980), and 67% (Van der Schaft et al., 1987). The PC distribution was examined by biochemical methods, utilizing covalent binding of membrane-impermeable reagents (Gordesky and Marinetti, 1973; Whiteley and Berg, 1974), enzymatic digestion (Verkleij et al., 1973), or use of phospholipid exchange proteins (Barsukov et al., 1976), but these methods are not appropriate for accurate measurement of asymmetry (Op den Kamp, 1979; Etemadi, 1980; Zachowski, 1993). Nevertheless, the divergent results from erythrocytes of different species were obtained by similar methods, suggesting that the PC distribution in non-human erythrocytes may not show such extreme asymmetry across the plasma membrane leaflet as that found in human erythrocytes.

More recently, freeze-fracture replica labeling EM utilizing metabolic labeling with a clickable choline analog indicated that PC exists in equivalent amounts in both leaflets of the plasma membrane in cells other than erythrocytes (Iyoshi et al., 2014). In contrast, studies applying an anti-PC antibody to freeze-fracture replicas showed predominant labeling in the outer leaflet, but the capture ratio was extremely low (Fujimoto et al., 1996; Murate et al., 2015), at least partially because the antibody preferentially detected only some PC subpopulations (Nam et al., 1990).

Although the exact ratio of PC in the two leaflets is not clear, the bulk of the data suggests that more than 20% of PC could exist in the inner leaflet of the erythrocyte membrane. In the plasma membrane of other cell types, the proportion of PC in the inner leaflet may be even higher.

### Aminophospholipids

Both PS and PE were initially thought to be confined to the inner leaflet of the human erythrocyte membrane, and a similar asymmetry was assumed to be a general property of all plasma membranes. Lack of cell surface binding of PS-specific annexin V (Koopman et al., 1994) and PE-specific Ro09-0198 (Emoto et al., 1996) in normal interphase cells further strengthened the assumption. However, the result with annexin V and Ro09-0198 only suggested that the level of PS and PE in the outer leaflet is below a threshold level, and cannot necessarily be equated with their complete absence. Actually, biochemical studies indicated that a sizable fraction of PS and PE exists in the outer leaflet, and moreover, that the proportion in the outer leaflet is highly variable among different cell types, 0–44% (PS) and 0–73% (PE) (it is generally higher for PE than PS; see tables in Devaux, 1991; Zachowski, 1993 for summary of results). Although the methods used in those studies may not be accurate or quantitative (Op den Kamp, 1979; Etemadi, 1980; Zachowski, 1993), it is difficult to explain the divergent results by the methodological insufficiency alone. We presume that non-negligible amounts of PS and PE distribute to the outer leaflet of the plasma membrane in most cells.

### Phosphatidylinositol and phosphoinositides

PI is also assumed to exist largely in the inner leaflet of the plasma membrane, but biochemical studies indicated that PI may also be present in the outer leaflet in the human erythrocyte membrane as well as in the outer plasma membrane leaflet of several nucleated cell types (Rawyler et al., 1985; Bütikofer et al., 1990; Gascard et al., 1991). The lack of a PI specific probe makes it difficult to confirm the above result microscopically, but several phosphoinositides could be labeled at the cell surface by applying membrane-impermeable probes (Gascard et al., 1991; Kale et al., 2010). This suggests that inositol phospholipids are present in the outer leaflet of the plasma membrane as physiological constituents.

### Sphingomyelin

The proportion of SM in the outer leaflet of the human erythrocyte membrane was reported to be 80–85% (Verkleij et al., 1973) and 79% (van Meer et al., 1981). In contrast to PC, erythrocytes of other species also showed a similar or even higher fraction of SM in the outer leaflet (Renooij et al., 1976; Crain and Zilversmit, 1980; Rawyler et al., 1985; Van der Schaft et al., 1987). Also in other cell types, the proportion of SM in the outer leaflet was generally shown to be higher than that of PC (see tables in Devaux, 1991; Zachowski, 1993 for summary of results). Nevertheless, it is notable that a significant proportion of SM, e.g., 10–20%, is found in the inner leaflet when biochemical methods are used. In addition, freeze-fracture replica labeling EM also showed labeling of the inner leaflet with an SM-binding toxin, lysenin (Murate et al., 2015). Quantitativity and specificity of the lysenin labeling in freeze-fracture replica needs to be rigorously tested, but the result is consistent with the presence of SM in the inner leaflet of the plasma membrane.

### Glycosphingolipids

Glycosphingolipids (GSLs) are generally believed to exist only in the outer leaflet. Glucosylceramide and galactosylceramide start their synthesis at the cytoplasmic side of the ER and the Golgi, and are then flipped to the luminal side for further glycosylations. Complex GSLs like gangliosides are not likely to flip back to the cytoplasmic leaflet because of their bulky hydrophilic headgroup, but short carbohydrate chain GSLs can translocate across the membrane (Buton et al., 2002). Therefore, the presence of a small amount of GSL in the inner leaflet of the plasma membrane cannot be excluded.

### Cholesterol

Despite the development of new methodologies (Frisz et al., 2013; Solanko et al., 2015), neither the lateral distribution of cholesterol nor its distribution across the two plasma membrane leaflets is well-characterized (Marquardt et al., 2015). A polyene antibiotic, filipin, has been used frequently to visualize endogenous cholesterol distribution both by fluorescence microscopy and EM. Filipin has been reported to probe also GM1 (Arthur et al., 2011), but given that 5–7 mol% cholesterol required for filipin visualization in membranes (Behnke et al., 1984) and the scarcity of GM1 this should not be a problem in cell staining. However, membrane deformation caused by filipin-cholesterol complex formation does not indicate in which leaflet of the membrane cholesterol exists. As an alternative, analogues with various fluorescent tags have been used, but none of them give satisfactory results, because the presence of large tags significantly changes the molecular property of cholesterol, in particular the quick flip-flop between the two leaflets (Klymchenko and Kreder, 2014; Sezgin et al., 2016).

Dehydroergosterol, that is very similar to cholesterol in the molecular structure, is considered to be the best fluorescent analog. Its optical properties are not favorable for imaging, but a study using dehydroergosterol showed its enrichment in the inner leaflet (Mondal et al., 2009), which is in accordance with theoretical considerations (Giang and Schick, 2014; Falkovich et al., 2016). In contrast, preferential distribution of cholesterol to the outer leaflet of the human erythrocyte membrane was shown by biochemical analysis of a phospholipid monolayer obtained by freeze-fracture (Fisher, 1976). It is not clear whether the disparity between the two studies is derived from difference between the cell types examined, methodology, or both.

### Acyl chains

The acyl chain composition of phospholipids is thought to be a critical factor to determine properties of individual membrane leaflets, but our knowledge of this aspect is even less complete than that of the head groups. Classical work using thin layer chromatography and phospholipase hydrolysis suggested that the proportion of saturated and unsaturated acyl chains is highly variable between different phospholipid species and also between different tissues (Yabuuchi and O'Brien, 1968; Wood and Harlow, 1969). Tandem mass spectrometric analysis confirmed the tissue-to-tissue variation, but it also indicated that PS, PE, and PI tend to have a higher proportion of polyunsaturated acyl chains than PC, which is enriched with mono- and di-unsaturated acyl chains (Hicks et al., 2006).

It is largely unknown how phospholipids with different acyl chain compositions distribute two-dimensionally in the plasma membrane. Notably, PC of particular acyl chain compositions were shown to have heterogeneous distributions along the neuronal axon both when assessed by imaging mass spectroscopy (Yang et al., 2012) and by use of a unique monoclonal antibody (Kuge et al., 2014). But with these methods, it is not easy to distinguish acyl chains in one leaflet of a membrane from another. Freeze-fracture may be one of a few possible ways to separate the two leaflets, but has not yet been used for analysis of the acyl chain composition.

### Ordered membrane nanodomains in the cytoplasmic plasma membrane leaflet

Currently the co-existence of two liquid phases, liquid ordered (lo)- and liquid disordered (ld), is the best explanation for domains of differential lipid packing observed in the plasma membrane although the perfect proof to characterize them as phases is almost as challenging to obtain as the absolute evidence for their dismissal. This neither means that proteins or lipids could not also show local enrichment unrelated to differential lipid packing, that plasma membrane domains need to be in the micron range nor that cellular membranes are at equilibrium (Ackerman and Feigenson, 2015; Mouritsen and Bagatolli, 2015).

An outstanding question in the membrane nanodomain field is whether lo-domains can form in the lipid mixes present in the inner leaflet of the plasma membrane. From model membrane studies it is clear that the lipid mixes mimicking the outer plasma membrane leaflet (PC, SL, and cholesterol), can form co-existing ld- and lo-domains (Ahmed et al., 1997; de Almeida et al., 2003; Veatch and Keller, 2005). The main phospholipids in the inner leaflet (PE, PS, and PC) can only form ld-phase even in the presence of cholesterol (Wang and Silvius, 2001; Kiessling et al., 2006). In fact, when the acyl chains of PE, PS, and PC are mostly unsaturated, as they are *in vivo*, lipid mixes with a high PE content do not even form a bilayer but form a hexagonal or cubic phase (Boni and Hui, 1983) and it was recently proposed that the high bending free energy of PE is what attracts cholesterol to the inner leaflet (Giang and Schick, 2014). Despite the failure of symmetric PE/PC/PS lipid mixes to sometimes form bilayers, they do make up one half of the cellular plasma membrane highlighting that effects of asymmetric lipid compositions cannot be predicted from studies of symmetrical model membranes. Moreover, the difference in the membrane order of lo- and ld-domains in most model membrane studies is far greater than that possible in the plasma membrane considering its lipid composition, making predictions of probe, lipid, and protein partitioning in the plasma membrane from such studies of limited use.

In model membranes lo-domains can be characterized biochemically by their insolubility in non-ionic detergents, Triton X-100 (TX) being the most widely used, that when used at 4°C produces detergent resistant membranes (DRMs) that float in sucrose density gradients. The amount of TX-DRMs reflects the fraction of lo-domains in the membranes (Ahmed et al., 1997) and the lipids retrieved in TX-DRMs can form lo-phase (Schroeder et al., 1994). In cells, the relationship between DRMs and lo-domains is more tenuous (Lichtenberg et al., 2005; Ashrafzadeh and Parmryd, 2015; Sevcsik and Schütz, 2016). TX-DRMs from T cells are gigantic rather than nanosize (Magee and Parmryd, 2003) and proteins normally resident in organelles other than the plasma membrane appear in T cell TX-DRMs (von Haller et al., 2001). However, TX-DRMs do suggest that lo-domains in the two plasma membrane leaflets are coupled since they are enriched in both sphingomyelin likely to originate from the outer leaflet and saturated glycerophospholipids probably originating from the inner leaflet relative to both total cell and plasma membrane lipids (Fridriksson et al., 1999). Moreover, TX-DRMs contain acylated proteins that in intact cells are anchored to the inner plasma membrane leaflet indicative of, but not evidence for, inner leaflet lo-domains (Melkonian et al., 1999).

Several other lines of investigation indicate that lo-domains could exist in the inner plasma membrane leaflet and highlight that leaflet asymmetry changes the behavior of monolayers. In molecular dynamics (MD) simulations of asymmetric bilayers, lo-domains in one leaflet can induce registered ordered domains in an opposing leaflet with a composition that on its own would not allow lo-domain formation (Perlmutter and Sachs, 2011; Polley et al., 2014). MD simulations and theoretical considerations have also suggested the opposite possibility that membrane fluidisation in one leaflet may cause a decrease in the order of or even prevent phase separation in the opposing leaflet (Wagner et al., 2007; Sun et al., 2015). In asymmetric supported bilayers, lo-domains on the supported side can induce lo-domains in the free side with inner leaflet lipid mixes that only form ld-phase on their own (Kiessling et al., 2006), a process that was later shown to require acyl chain diversity among the inner leaflet lipids (Wan et al., 2008). In black membranes it has also been demonstrated that ld and lo phase separation in one leaflet can induce phase separation in an opposing ld-phase lipid mix and moreover that ld and lo phase separation can be suppressed by ld-phase lipid mixes (Collins and Keller, 2008). Also in asymmetric vesicles, produced by lipid exchange, registered lo-domains can be induced in a ld lipid mix by an opposing ld and lo phase separated bilayer (Lin and London, 2015) and, conversely, a gel phase becomes less ordered than that in symmetric bilayers when facing ld-phase (Heberle et al., 2016). However, direct evidence of the existence of inner plasma membrane leaflet lo-domains is still missing.

## The registration of membrane nanodomains in the two leaflets

**Figure 2:**
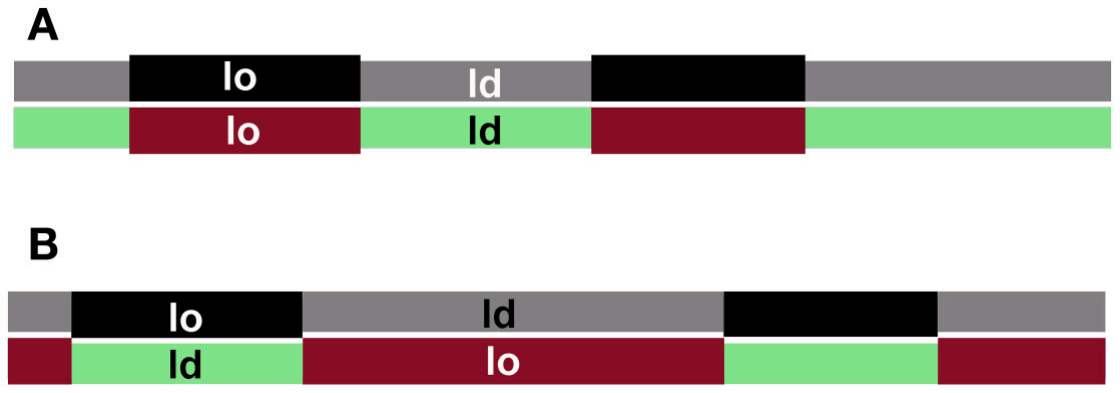
**Domain registration across the bilayer**. Interleaflet interactions favor domains in the two leaflets to be registered **(A)**, whereas hydrophobic mismatch favors domain antiregistration **(B)**, which serves to even the membrane thickness.

The aggregation of registered domains containing non-transmembrane signaling molecules could facilitate signal transduction across the plasma membrane by separating deactivating molecules from their substrates (Simons and Toomre, 2000; Magee et al., 2005; Mongrand et al., 2010), but whether domains in cell plasma membranes are registered is largely unknown. When ld- and lo-domains co-exist in both leaflets of a bilayer, they can adopt two strategies to minimize the energy cost of the domain thickness mismatch. Domains could either be registered to minimize the contact area between the two phases, or anti-registered to minimize the difference in thickness over the bilayer (Figure 2). Note that complete anti-registration is only possible when each domain type occupies 50% of the total membrane distributed at any fractional division between the two leaflets. Since the proportion of plasma membrane lo- and ld-domains in live cells is not fixed (Mahammad et al., 2010; Owen et al., 2012; Dinic et al., 2013; Golfetto et al., 2015), domain registration is the more likely scenario. Moreover, the likely similarity in lipid composition of plasma membrane ld- and lo-phases favors their registration (Fowler et al., 2016).

Contributing factors to domain registration could be transmembrane proteins, lipid interdigitation, membrane curvature, line tension, cholesterol flip-flop, and electrostatic interactions (Nickels et al., 2015) of which chain interdigitation has been invoked as the major contributing factor (May, 2009) but also has been totally dismissed (Collins, 2008). A recent experimental determination of the strength of the coupling parameter that causes domain registration matches a theoretical predictions with values around 0.01 *k*_*B*_*T*/nm^2^ (Putzel et al., 2011; Blosser et al., 2015), but considerably higher values have also been suggested from theoretical considerations (May, 2009). Intriguingly, MD simulations of polymers have shown that forced splitting of registered lo-domains make them move toward one another long before they are in contact, i.e., they sense each others presence through the ld-phase (Pantano et al., 2011). This suggests that coupling between lipids is substantial and in symmetric bilayers the energy involved in the coupling has been estimated to be around 100 cal/mol of phospholipid (Zhang et al., 2007).

Recently, it has been argued both that line tension alone (Galimzyanov et al., 2015) or the competition between line tension and leaflet coupling determines domain registration (Williamson and Olmsted, 2015). However, line tension in the plasma membrane is likely to be small due to the wide range of lipid species available to smoothen any hydrophobic mismatch—a diversity that would also result in a high similarity in the composition of co-existing ld- and lo-domains. Thus the lipid diversity and as well as the asymmetric lipid distribution need to be incorporated into both theoretical and model membrane studies. In their absence the results are less physiologically relevant.

Registration of lo-domains in asymmetric bilayers has been described in MD simulations, supported bilayers, black membranes and vesicles (Collins and Keller, 2008; Wan et al., 2008; Cheng and London, 2011; Chiantia et al., 2011; Perlmutter and Sachs, 2011; Polley et al., 2014), indicating that registration is the favored organization also in cells. Of note is that domain coupling in supported bilayers is dependent on distancing the membrane from the support (Garg et al., 2007).

We pioneered cell studies on the registration of plasma membrane nanodomains between leaflets by using a probe that exclusively report on the membrane order of the outer plasma membrane leaflet. We found that decreasing the level of actin filaments attached to inner plasma leaflet lipids resulted in a lower fraction of lo-domains in the outer leaflet (Dinic et al., 2013). The implication of our results is that a direct effect on one plasma membrane leaflet is communicated to the other without the involvement of transmembrane proteins.

## Size of domains

When interleaflet coupling is studied in liposomes and supported lipid bilayers, domains of 1 μm or larger are usually examined, but such large domains are not likely to exist in the plasma membrane of most mammalian cells (Lingwood and Simons, 2010; Ashrafzadeh and Parmryd, 2015). Actually, studies using single particle tracking indicated that a transient domain as small as 10 nm can transmit signals to the cytoplasm to induce physiological reactions (Suzuki et al., 2007a,b). Therefore, in order to study physiological importance of domain registration in the plasma membrane, domains in the small size range need to be examined.

This is a challenging task, however, considering the methods currently available. For example, GFP-tagged lipid-binding proteins are commonly used as probes to define the lipid distribution by fluorescence microscopy. But when one probe molecule binds to the head group of a target lipid, which should be in the range of ~1 nm in diameter, binding of another probe molecule to adjacent target lipids is precluded due to steric hindrance. This limitation is not derived from the limitation in spatial resolution of microscopes, but from the large size of probes compared to lipid head groups, so that use of super-resolution microscopes is not helpful (for other potential problems of the method, see Takatori et al., 2014). Interferometric detection of scattering circumvents several of the problems of particle tracking and has a lateral resolution in the low nm scale (Lindfors et al., 2004), but has not yet been applied to cells. Importantly tracking methods can, if cell topography is accounted for (Adler et al., 2010), detect anomalous diffusion caused by plasma membrane domain co-existence but do not reveal what is causing domain formation; for example differential lipid packing, protein concentration or charge aggregation.

In electron microscopy, colloidal gold particles of larger than 5–10 nm in diameter are often used as markers and one such gold particle may overlay many lipid molecules (Fujita et al., 2007; Zhou et al., 2014). Therefore, a cluster of colloidal gold labels indicates enrichment of the target lipid in the area, but does not necessarily indicate clustering of lipid molecules (although clustering of lipids is likely to occur more frequently when their local density is higher).

It is even more difficult is to examine whether domains in the two leaflets are registered. Use of complementary freeze-fracture replicas has enabled the examination of matching areas in the two leaflets (Hagiwara et al., 2005), but even with this method, the registration of small domains is difficult to study without a technical breakthrough.

## Crossing the mid-line—a privilege of asymmetric lipids with one long acyl chain

**Figure 3:**
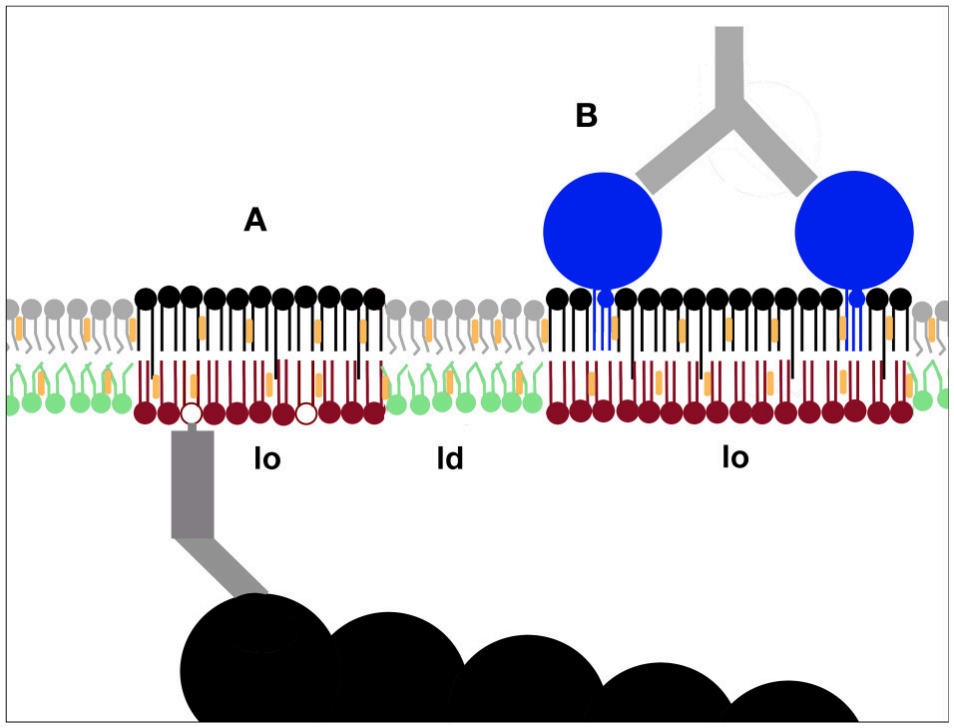
**Pinning as a mechanism to nucleate membrane nanodomains**. Immobilising plasma membrane components can induce lo-domain formation. This has been demonstrated both for inner leaflet phoshoinositides linked to actin filaments **(A)** and outer leaflet components like GPI-anchored proteins **(B)** (Dinic et al., 2013). The creation of new lo-domains is likely to play an important role in the molecular sorting required for cellular processes such as signaling and membrane trafficking.

The lipid raft hypothesis states that small tightly packed lipid domains float in a sea of less tightly packed lipids and proposes that lipid rafts in the two leaflets are coupled by the interdigitation of long saturated acyl chains on glycosphingolipids in the outer leaflet extending into the inner leaflet (Simons and Ikonen, 1997). The proposed type of interdigitation was different from the initial use of the word for long acyl chains spanning the entire hydrophobic core of the bilayer made up of lipids with considerably shorter acyl groups (Boggs and Koshy, 1994; Schram and Thompson, 1995). Instead the now accepted meaning of interdigitation, long acyl chains crossing the midplane of the bilayer and penetrating a small distance into the opposing leaflet (Figures 1, 3), was highlighted. This type of interdigitation is for instance observed for long acyl chain (C24) glycosphingolipids when they are minor components in a biologically relevant (C16-C18) matrix (Mehlhorn et al., 1988; Morrow et al., 1995). Although the ideas brought together in the lipid raft hypothesis were not new, it had an enormous impact in promoting membrane research. Much of the polarized discussion in the field has been caused by the use of questionable methodology (Klotzsch and Schütz, 2013; Ashrafzadeh and Parmryd, 2015), but the existence of plasma membrane nanodomains with different lipid packing was suggested from studies that predate the lipid raft hypothesis by decades (Morrisett et al., 1975; Karnovsky et al., 1982). Although frequently misinterpreted, the 1972 fluid mosaic model does not rule out, but rather insists, that short-range order in the plasma membrane exists (Singer and Nicolson, 1972), an important feature that was recently clarified by one of the model founders (Nicolson, 2014).

Sphingolipids (SLs) can have a big difference in chain length between the sphingosine chain, that tends to be C18, and the amide-linked acyl chains, that differ from C16 to C24 in naturally occurring SLs, creating many asymmetric SL species. Unsurprisingly, MD simulations of symmetric bilayers have shown that the greater the length mismatch between the matrix lipid acyl chains and the greater the length mismatch between the acyl and the sphingosine chains, the more interdigitation is observed (Niemelä et al., 2006). Bending of long acyl chains toward their own leaflet is also a possibility and has recently been observed in both MD simulations of SLs and model membranes studies of free fatty acids in glycerophospholipid bilayers (Paz Ramos et al., 2016; Róg et al., 2016). However, bending toward the water interface can be greatly reduced by cholesterol revealed by a model membrane study on methyl distributions (Mihailescu et al., 2011). Interestingly, simulations show that coupling is stronger if there is more cholesterol in the outer than in the inner leaflet (Róg et al., 2016).

A recent simulation study on symmetrical membranes made up of asymmetric PCs with differences in their acyl chain lengths suggested that lipid complementarity in chain length could act to assure uniform acyl chain packing (Capponi et al., 2016). This is along the lines of previous lipid complementarity studies indicating that, when present at matching concentrations, short acyl chains are found opposite long ones to create a smooth membrane (Zhang et al., 2004, 2005; Stevens, 2005). That such an organization should occur in biological membranes, with diverse and asymmetric lipid compositions, is less clear, especially since domains of different order and thus presumably thickness are found in the plasma membrane (Gaus et al., 2003; Dinic et al., 2013).

That asymmetric lipids from both leaflets can affect coupling in asymmetric membranes was first demonstrated in GUVs where SM C24:0 was shown to reduce the diffusion of POPC far more than that of DOPC (Chiantia and London, 2012). A recent MD simulation study, with asymmetric membranes with compositions closely mimicking cell plasma membranes, supported this finding with another lipid class and found a stronger coupling firstly in asymmetric vs. symmetric membranes and secondly the strongest coupling when the inner leaflet contained PS 18:0/18:1 and the outer leaflet contained SM 24:0 (Róg et al., 2016). That PS 18:0/18:1 plays an important biological role was implied in a recent study revealing that PS is required for retaining cholesterol in the inner leaflet in cells and that PS 18:0/18:1 uniquely has the capacity to protect cholesterol in model symmetrical membranes from oxidation (Maekawa and Fairn, 2015). Intriguingly, GPI-anchor protein clustering was also shown to be dependent on inner leaflet PS but with long acyl chains, rather than a specific acyl chain combination, being the requirement (Raghupathy et al., 2015). This was interpreted as enabling interaction with the long acyl chains required for the GPI-anchored proteins to cluster allowing cross-talk of specific lipids across the bilayer midplane. In light of the study discussed above (Maekawa and Fairn, 2015), we speculate that the PS may have been required to maintain the cholesterol level in the inner leaflet at a level sufficient for the formation of lo-domains since clustered GPI-anchored proteins are found in such (Dinic et al., 2013) and cholesterol previously has been identified as important for their clustering (Sharma et al., 2004).

## Imperfection in lipid asymmetry and domain registration

As discussed above, lipids have asymmetric distributions in the plasma membrane, but the asymmetry is not complete and non-negligible amounts of most lipids are likely to exist in the less-favored leaflet. Such imperfection in asymmetry, especially that of SM, could be of considerable importance for domain registration. In model membranes interleaflet coupling can occur with no (or a minimal amount of) SM in the inner leaflet (Wan et al., 2008; Visco et al., 2014; Lin and London, 2015). However, if a small but significant amount of SM exists the inner leaflet, as suggested by biochemical and histochemical studies (Devaux, 1991; Zachowski, 1993; Murate et al., 2015), that SM could potentially contribute to lo-domain formation, which could make coupling with the outer leaflet lo-domains very efficient (Halling et al., 2008; Lönnfors et al., 2011). Naturally this SM-SM interaction mechanism does not exclude participation of other phospholipids in lo-domain formation and interleaflet coupling. Rather, the SM-based coupling may function as an initial seed to induce further changes by lateral interaction with other phospholipids, such as PS or inositol phospholipids.

## Pinning

It has been shown repeatedly that the behavior of outer leaflet lipids and GPI-anchored proteins is influenced by actin dynamics (Fujiwara et al., 2002; Kwik et al., 2003; Goswami et al., 2008; Fujita et al., 2009; Mueller et al., 2011), but how actin in the cytoplasm can exert effects on the outer leaflet of the plasma membrane has been a longstanding enigma. Cell topography and the problematic interpretation of three dimensional data in 2D could explain at least some of this behavior (Adler et al., 2010), but pinning is also important for the communication between actin filaments and the outer leaflet.

Pinning, i.e., when the mobility of membrane components is either reduced or absent, alters the mixing entropy, which can alter the phase behavior and/or membrane organization (Putzel and Schick, 2009; Arumugam and Bassereau, 2015). Protein pinning can transmit the rearrangement of proteins from one leaflet to the other. This was first studied in erythrocyte ghosts where it was shown that the enclosure of antibodies to spectrin, a protein associated with the inner leaflet, caused the rearrangement of glycolipids and glycoproteins in the outer leaflet (Nicolson and Painter, 1973). Analogously, binding of lectins to the outer leaflet caused the rearrangement of spectrin (Ji and Nicolson, 1974). Lectins are also known to affect the physical properties of the plasma membrane (Evans and Leung, 1984). For lipids, pinning can nucleate either an ld-phase as shown in phosphatidylinositol 4,5-bisphosphate [PI(4,5)P2]-containing giant unilamellar vesicles (GUVs) where actin polymerisation induced phase-separation (Liu and Fletcher, 2006), an lo-phase as shown in GUVs by GM1 cross-linking (Hammond et al., 2005) or upon adhesion (Gordon et al., 2008).

Simulations on critical point fluctuations have indicated that pinning of lipids to actin filaments could prevent large-scale phase separation (Ehrig et al., 2011; Machta et al., 2011) and earlier simulations suggested that membrane protein obstacles or pinning could have the same effect (Yethiraj and Weisshaar, 2007; Fan et al., 2010; Gómez et al., 2010), phenomena that were recently observed in model membranes (Honigmann et al., 2014; Arumugam et al., 2015). Critical point fluctuations may however be superfluous for phase separation since it can be achieved by coupling of mechanical forces like actin polymerisation to membrane composition (Sens and Turner, 2011). That pinning does prevent large-scale phase separation in the plasma membrane is supported by the observation that visible large-scale phase separation can be seen in giant plasma membrane vesicles (GPMVs) that do not contain cortical actin (Sengupta et al., 2008; Levental et al., 2011). Furthermore, membrane blebs in live cells, where the membrane connection to actin filaments has been lost (Charras et al., 2005), are structures in the μm range with a lower laurdan generalized polarization-value than that found in the bulk plasma membrane of the same cells, indicative of disordered lipid arrangement (Dinic et al., 2013). Since lo-domains form where actin filaments are pinned to the plasma membrane, blebs may contain ld-phase only and represent an example of microscopically visible domains in live cells alongside the remaining intermittently actin filament pinned, and from a lipid packing perspective, more heterogeneous plasma membrane of live cells.

In asymmetric model membrane studies the general assumption is that an outer-side-like lipid mix could induce domain formation in a cytoplasmic-side-like lipid mix that cannot phase separate on its own (Kiessling et al., 2006; Collins and Keller, 2008; Lin and London, 2015) but the opposite is less frequently considered. However, in cells phosphoinositides in the inner plasma membrane leaflet can be pinned to intracellular actin filaments and cause the formation of lo-domains in the outer plasma membrane leaflet (Figure 3A), demonstrating that there is an inside out communication for the lipid packing of the two leaflets (Dinic et al., 2013). Lo-domains can also form when outer leaflet components like GM1 and GPI-anchored proteins are patched (Figure 3B), demonstrating that the physical state of the plasma membrane is also influenced by the cell's extracellular environment (Dinic et al., 2013). That immobilization by pinning can cause the formation of lo-domains in live cells was thus demonstrated in 2013 (Dinic et al., 2013). Despite this a later prominent study using simulations and GPMVs concluded by speculating that pinning induced lo-domain formation might be possible in live cells, claiming it as a novel and original idea, reversing the usual process of observation following speculation (Raghupathy et al., 2015).

A link between GM1, pinning and interdigitation has recently been found in both simulations and model membrane studies (Spillane et al., 2014; Sun et al., 2015). It seems likely that the lo-domain formation observed in the plasma membrane after cross-linking outer leaflet components, described above, causes the pinned molecules to stretch and hence make contact with the inner leaflet lipids, both nucleating and stabilizing the domains. Pinning may also result in membrane curvature that affects both leaflets (Deverall et al., 2008). Although we are well-aware of both the importance and prevalence of membrane curvature in cells (Adler et al., 2010; Parmryd and Onfelt, 2013), we have chosen to limit the discussion in this review to other mechanisms of importance for membrane nanodomain formation.

PI(4,5)P2 is known to interact with many actin-binding proteins, regulating their activity and localization (Saarikangas et al., 2010) and may be the phosphoinositide that links changes in actin dynamics to the proportion of plasma membrane lo-domains (Dinic et al., 2013). Interestingly, although PI(4,5)P2 often contains a polyunsaturated acyl chain (McLaughlin et al., 2002), which might not be expected to be found in lo-domains, PI(4,5)P2 is nevertheless enriched in plasma membrane lo-domains (Parmryd et al., 2003). A likely explanation for this apparent paradox is that the expectations arise from the behavior of lipids in well-separated ld- and lo-domains in model membranes that have an order difference impossible to achieve in the plasma membrane.

PS is also a candidate to mediate the actin-outer leaflet lipid coupling, as it is known to bind numerous cytoplasmic proteins (for a review, see Stace and Ktistakis, 2006), including actin-binding proteins (Cohen et al., 1986; Muguruma et al., 1995; Makuch et al., 1997). Actually, the mobility of PS in the plasma membrane has been shown to increase upon actin depolymerization (Kay et al., 2012; Zhou et al., 2014), indicating that PS may be constitutively bound to the actin meshwork or its movements confined by topographical features of the membrane (Adler et al., 2010). Binding between PS and actin-linking proteins is thought to occur electrostatically to the PS head group and thus should be independent of the acyl chain composition (Wood and Harlow, 1969; Hicks et al., 2006), but it is likely to be enhanced if PS is clustered.

Interestingly, the acyl chain composition of the different phospholipid species, especially PS, varies between tissues (Hicks et al., 2006). Both the efficiency of domain registration and interleaflet coupling may differ with acyl chain composition: with a stronger coupling, the inner leaflet should become more ordered and hence similar to the outer leaflet. How the acyl chain composition is regulated is not clear, but Land's cycle, remodeling inner leaflet phospholipids (Hishikawa et al., 2014), may be involved. The regulatory mechanism and the possible correlation between the acyl chain composition and interleaflet coupling warrant further studies.

## Outlook

Compared with amino acids and nitrogenous bases the lipid diversity is enormous and intriguing. Similarly, compared to proteins and nucleic acids, our knowledge of membranes is limited. Tools are now being developed to for instance unravel the roles of individual lipid species in cellular processes, to study the effect of leaflet asymmetry and to perform detailed examination of the lipid distribution within leaflets—all outstanding questions which require close collaborations of scientists from physical, chemical, and biological disciplines. We predict that within the next decade exciting interdisciplinary breakthroughs in the membrane biology field will unravel mechanisms of fundamental biological processes and that we will approach a consensus regarding the nature of plasma membrane nanodomains.

## Author contributions

IP conceived the idea. IP and TF wrote the review.

### Conflict of interest statement

The authors declare that the research was conducted in the absence of any commercial or financial relationships that could be construed as a potential conflict of interest.
